# Review of systemic sclerosis and antineutrophil cytoplasmic antibody vasculitis overlap: Using autoantibodies for a personalised medicine approach

**DOI:** 10.1177/23971983221126850

**Published:** 2022-09-30

**Authors:** Kristina EN Clark

**Affiliations:** UCL Centre for Rheumatology, University College London, London, UK

**Keywords:** Systemic sclerosis, ANCA vasculitis, overlap disease, antibodies, scleroderma

## Abstract

Both antineutrophil cytoplasmic antibody-associated vasculitis and systemic sclerosis are rare autoimmune diseases. Both have the potential for significant multi-organ involvement, and both carry high morbidity and mortality. Disease-specific autoantibodies in these conditions allow for risk stratification for organ-based complications, and for personalised therapeutic strategies. The concomitant presentation of antineutrophil cytoplasmic antibody-associated vasculitis and systemic sclerosis is rare, and only reported in up to 1.3% of systemic sclerosis cases. These patients present more frequently with anti-myeloperoxidase and anti-topoisomerase antibody profiles, with increased incidence of interstitial lung disease and renal involvement than would be expected in either disease independently. Appreciating the role of the autoantibodies in each disease state, and where they overlap, allows for the potential of a more personalised approach to managing these complex patients.

## Introduction

Autoantibodies not only facilitate the diagnosis of autoimmune diseases, but also allow for risk stratification of potential future complications. Few diseases exhibit this better than in systemic sclerosis (SSc), and antineutrophil cytoplasmic antibody (ANCA)-associated vasculitis (AAV).

Scleroderma-overlap syndrome refers to patients who satisfy the criteria for SSc together with clinical or laboratory features of another autoimmune rheumatic disease. Up to 20% of SSc patients fulfil these criteria, with polymyositis/dermatomyositis being the most common.^1^ SSc-AAV overlap is rare, with a prevalence of up to 1.6% in SSc patients.^2^

There are a handful of case reports focussing on large- and medium-vessel vasculitis overlap with SSc, the commonest, yet still rare, overlap is with small-vessel vasculitis, thus, the focus of this review. Both these conditions are predominated by unique autoantibody profiles, and these antibodies confer differing risk for organ complications. The aim of this review is to highlight the impact-specific autoantibodies have in the context of these rare autoimmune diseases individually, and when identified concomitantly.

Vascular abnormalities dominate in both SSc and AAV, through different underlying pathologies. In SSc, vasculopathy is one of the earliest features, and results in the development of Raynaud’s phenomenon, digital ulcers and serious complications, such as pulmonary arterial hypertension (PAH). In AAV, small-vessel inflammation is more common, typically associated with respiratory tract and kidney involvement.

AAV is a rare group of diseases with multi-organ complications, characterised by small-vessel inflammation. It comprises predominantly of three major subtypes: granulomatosis with polyangiitis (GPA), microscopic polyangiitis (MPA) and eosinophilic granulomatosis with polyangiitis (EGPA). Like SSc, it is associated with autoimmunity, and has disease-specific ANCAs associated with it. These are largely mutually exclusive, namely, peripheral (p-ANCA) or cytoplasmic (c-ANCA), each associated with differing protein targets, respectively, anti-MPO (antibodies against myeloperoxidase) and anti-PR3 (proteinase 3).

AAV has an overall incidence of 13–20 per million people, and a prevalence of 300–421 per million persons.^3^ Rates are similar in SSc, with an annual incidence of 10–20 per million persons, and prevalence of 100–250 per million.^4^ SSc is a female predominant disease, with a female to male ratio between 3:1 and 8:1, whereas, AAV demonstrates equal sex distribution.^3^

Both these two diseases are associated with premature mortality, significant morbidity and poorer quality of life.^3,5^

There is well established geographical variation between the subtypes of AAV. PR3-ANCA positivity increases with increasing latitude. MPO-ANCA vasculitis is more frequently identified in patients from Japan compared to the United Kingdom, as well as in Chinese and Southern European populations.^6^ Patients with PR3-ANCA are more likely to have a diagnosis of GPA (65% of GPA patients have a positive anti PR3 antibody), whereas those with the MPO-ANCA antibody tend towards features of MPA (55% of MPA patients).^7^

AAV and SSc are both characterised by hallmark autoantibodies, which are largely mutually exclusive.^8^ These antibodies confer differing risk of organ involvement, pattern of organ involvement, and more recently therapeutic strategy within the disease.

The three commonest hallmark autoantibodies in SSc (found in 60%–80% of patients) include anti-topoisomerase antibody (ATA, anti-Scl-70), anti-RNA polymerase III antibody (ARA), both found more frequently in diffuse cutaneous SSc (dcSSc) (20%–30% of SSc patients, (85% dcSSc) and 10%–20% of SSc patients (> 80% dcSSc), respectively), and anti-centromere antibody (ACA), found in up to 40% of patients (95% of them being classed as limited cutaneous SSc (lcSSc)).^8,9^

## Clinical phenotypes by autoantibody subtypes within the individual disease

The relationship between ANCA subtype and clinical manifestations within ANCA vasculitides is increasingly a focus of research; an area also explored within SSc. Traditionally, SSc subclassification has mainly focussed on the extent of skin involvement (diffuse vs limited), but recently, the combination of autoantibody subtype and extent of disease allows for more specific risk stratification.^9^

ATA+ patients carry a significantly higher risk of developing interstitial lung disease (ILD) within the first 5 years of disease onset (up to 80%), whereas patients with ARA + have a much lower risk of ILD (33%), but the highest risk for scleroderma renal crisis (SRC) (28% at 20 years) compared to other autoantibody subtypes^9^ (Table 1). Even within one autoantibody subtype, extent of skin involvement can determine differing risk. For example, patients with ATA + dcSSc have the lowest survival at 20 years (32.4%), whereas ATA + lcSSc have a significantly higher survival rate at 20 years (61.8%).^9^

**Table 1.** Organ involvement distribution based on autoantibodies in SSc and AAV alone, and in SSc-AAV combined.^10^

	SSc-ATA-positive	SSc-ARA-positive	SSc-AAV	AAV-MPO-positive	AAV-PR3-positive
Antibody			58%–88% ATA. Majority patients MPO		
Prevalence in disease	20%–30%	10%–20%	1.6% of SSc patients	Increased positivity in Asia and Southern Europe	Increased prevalence in Northern Europe and the United States
Disease subgroup	85% dcSSc	> 80% dcSSc	50% lcSSc	> 90% MPA	90% GPA
**Organ involvement**					
Renal	4.6% SRC	28 % SRC	75%–80% – majority pauci-immune glomerulonephritis/rapidly progressive glomerulonephritis	80% with more fibrotic changes (19%–25%	80%. More normal glomeruli seen fibrotic features in 10%–15%
Lung	70% ILD	33% ILD	80% ILD	46%–71% ILD (fibrosing lesions)	0%–29% ILD (cavitating lesions with more alveolar haemorrhage recorded)
Cutaneous involvement	> 95% develop skin fibrosis, typically in the diffuse distribution	> 95% develop skin fibrosis, typically in the diffuse distribution	More frequently lcSSc skin distribution; > 10% develop necrotizing vasculitis or vasculitic skin rash on top of scleroderma skin involvement	20%–40% vasculitic skin rash	20%–40% vasculitic skin rash
Neurological involvement	< 18%, majority carpal tunnel	< 18%, majority carpal tunnel	15%	7% mononeuritis multiplex, peripheral neuropathy and myopathies	7% mononeuritis multiplex, peripheral neuropathy and myopathies

ATA: anti-topoisomerase; AAV: antineutrophil cytoplasmic antibody-associated vasculitis; PR3: proteinase; MPA: microscopic polyangiitis; GPA: granulomatosis with polyangiitis; SRC: scleroderma renal crisis; ILD: interstitial lung disease.

The clinical phenotype differs by autoantibody subtype in AAV, with PR3-ANCA+ patients mainly suffering ear, nose, sinus and throat involvement. MPO-ANCA+ patients have an increased risk of renal disease compared to PR3-ANCA disease, with associated increased severity and chronicity.^7^ Even within patients who are MPO-ANCA+, those with a diagnosis of MPA are more likely to have significant renal involvement compared to the GPA phenotype.

There are subtle organ-based differences between the antibody subtypes in GPA. PR3-ANCA + GPA patients are more likely to have severe organ involvement compared to MPO-ANCA + GPA patients,^11^ and a greater need for aggressive immunosuppression.

The prevalence of ILD is not uniform throughout the AAVs. ILD is more frequently diagnosed in patients with MPA (45%) compared to GPA (23%).^12^ The vast majority of ILD cases are associated with anti-MPO (46%–71%), with anti-PR3 only being present in 0%–29% of cases. The distribution of lung involvement also differs by antibody subtypes (Table 1). Central airways disease, with cavitatory lesions and/or nodules are more prevalent in PR3-ANCA+ patients,^11,13^ whereas, MPO-ANCA+ patients are more likely to have usual interstitial pneumonia (UIP) pattern ILD or bronchiectasis. These MPO-ANCA patients with bronchiectasis have an increased risk of peripheral nerve involvement and a reduced frequency of renal involvement.^13,14^

Subgroup analysis is increasingly highlighting differing treatment response by autoantibody. ATA-positive patients showed a greater response to tocilizumab, with a greater stability in lung function compared to other autoantibody subgroups.^15^ In contrast, the clinical trial of riociguat showed greater benefit in preventing skin progression (as measured by the modified Rodnan skin score (MRSS)) in the ARA subgroup,^16^ while nintedanib demonstrated a numerically greater preservation of lung function in the ATA-negative cases compared to the ATA-positive cases.^17^

Emerging data within the ANCA vasculitis field has suggested, for severe cases, PR3-ANCA+ patients respond better to rituximab, whereas, MPO-ANCA+ patients tend to respond better to avacopan (anti-C5a receptor).^18,19^

### SSc and AAV in overlap

#### Epidemiology

The proportion of patients with SSc and a positive ANCA, MPO or PR3 is significantly higher than the proportion of patients with an active vasculitis. Up to 12% of patients with SSc are ANCA+.^20–22^ The most common ANCA specificity identified in SSc-AAV is consistently p-ANCA with anti-MPO in 5.9%–8% of SSc patients,^21,22^ while c-ANCA positivity is rare. Anti-PR3 and anti-MPO antibodies have both been reported in SSc patients, with up to 22% being anti-MPO+,^21,23^ and significant overlap with p-ANCA positivity. The Australian scleroderma study identified anti-PR3 in 13.8% of patients, compared to 11.2% with anti-MPO,^22^ with rare reports of dual positivity.^21^ The discrepancy has been postulated to arise from the methods of testing for the ANCA, whether done by enzyme-linked immunosorbent assay (ELISA) or by indirect immunofluorescence (IIF).^10,22^

Within the ANCA + SSc patient cohort, those that are anti-MPO + had a higher frequency of ATA positivity compared to those that were not anti-MPO + .^22^ However, the frequency of ANCA, anti-MPO or anti-PR3 in SSc is likely to be lower than reported, as ANCA testing is not routine in asymptomatic patients

The true prevalence of clinically significant AAV in SSc is around 1.6%.^2^ Patients with SSc-AAV are more likely to be female,^23^ and more likely to be lcSSc (especially with MPO-ANCA vasculitis).^2,22^ There is increased prevalence of ATA in the overlap cohort (58.8%–77.7% compared to 20%–30% in SSc alone^9^), but other associated antibodies include anti-U1 RNP and anti-U3 RNP.^2^ However, given the rarity of SSc-AAV, the absolute numbers are still extremely low. Historically, penicillamine was associated with a reversible membranous glomerulonephritis in patients with SSc-AAV, but is now largely seen as a historical trigger, given this treatment is no longer used in SSc.^23,24^

## Pathogenesis

There are overlapping features in the pathogenesis of SSc and AAV, which may contribute to the serious complications of concomitant diagnosis, most specifically seen with vascular features, with vascular injury being a prominent feature of both diseases.

Small-vessel vasculopathy is one of the earliest manifestations of SSc, presenting as Raynaud’s phenomenon. Through an ischaemia/reperfusion process, endothelial cell activation leads to an increase in adhesion molecules, promoting leukocyte recruitment, proliferation of vascular smooth muscle cells and activating fibroblasts. Subsequent cell injury results in abnormal repair of blood vessels, vascular remodelling, intimal proliferation of arterioles and capillary breakdown, resulting in dysregulated angiogenesis, capillary loss and dilatation.^25,26^

AAVs affect small-to-medium vessels. ANCAs are directed against enzymes found within primary granules of neutrophils and lysosomes in monocytes. Unlike in SSc, the ANCA are pathogenic and implicated directly in the development of small-vessel vasculitis.^27^ ANCA can directly bind to MPO or PR3 expressed on the surface of primed neutrophils, and engage Fc receptors. This results in the release of proteolytic enzymes and reactive oxygen species, as well as neutrophil extracellular traps (NETs). The NETs result in direct injury to the endothelium, activation of the immune system and complement cascade. C5a activation leads to further priming of neutrophils,^28^ predisposing to endothelial injury and ultimately necrotizing vasculitis, with fibrinoid necrosis of the vessel wall.

However, the discrepancy between positive antibodies, and clinical manifestations in SSc-AAV highlights that ANCA positivity is not always pathogenic. One theory suggests that the ANCA present in SSc may have poor affinity and avidity for the epitope, making it less pathogenic. In SSc, the pathogenicity of autoantibodies is not as clear. Whereas, there is evidence that ATA is pathogenic on fibroblasts and endothelial cells, other antibodies, such as ARA may more be markers of pathology.^29^

## Genetics

A large genome side study in SSc confirmed associations with human leukocyte antigen (HLA)-DRB1*11:04 and HLA-DPB1*13:01. Specific associations of HLA-DQA1 allele were identified with lcSSc or dcSSc.^30^

In ANCA vasculitis, the HLA-DP had stronger associations with PR3-ANCA, while MPO-ANCA was significantly associated with and HLA-DQ SNPS.^31^ Both GPA and MPA are closely associated with major histocompatibility complex (MHC) Class II genes, but with clear distinctions between the two.

There is evidence of shared HLA haplotypes in patients with SSc-AAV, supporting a genetic susceptibility to fibrosis.^2^

## Clinical features

Both SSc and AAV individually are multi-organ diseases, affecting primarily the kidneys and the lungs. Therefore, unsurprisingly, the overlap of these two conditions can result in serious internal organ complications. Patients typically present with features of MPO-ANCA + MPA phenotype, or a renal-limited vasculitis compared to a GPA presentation.^22,32^

### Renal involvement

The majority of renal presentations are consistent with a diagnosis of MPA (typically an MPO-ANCA vasculitis), or renal-limited vasculitis,^22,23,32^ with only one case of GPA in a patient with SSc.^2^

Renal involvement is almost universal in patients with SSc-AAV, affecting 75%–80% of both lcSSc or dcSSc patients.^23,32^ It is important to differentiate a presentation of acute vasculitis of the kidney from a presentation of SRC due to differing approaches to management. If AAV is the driver of the renal disease in SSc-AAV, the onset tends to be later than SRC (median 7 years compared to 1–4 years), subacute in presentation, with patients being normotensive or mildly hypertensive, and an active urinary sediment (heavy proteinuria and haematuria). This contrasts to SSc patients presenting with SRC, where presentation involves acute onset malignant hypertension and mild proteinuria (< 1 g/day).^10,33^ In one review, only 1/3 of patients with SSc-AAV were hypertensive, and none showed any evidence of malignant hypertension leading to end-organ damage. Microangiopathic haemolytic anaemia was seen in 50% of SSc-AAV patients presenting with SRC.^23^

Renal biopsies frequently show pauci-immune glomerulonephritis with glomerular crescent formation,^23,32^ unlike in SSc, where the histological changes involve thrombotic microangiopathy, with proliferative obliterative vasculopathy and myxoid intimal changes, onion-skin narrowing of arterioles and fibrointimal sclerosis.^2^ Occasionally, SSc-AAV patients have features of arcuate arteritis, which is more commonly seen in SRC. Patients with SRC are more frequently ARA-positive, unlike in SSc-AAV where ATA dominates antibody profiles.

### Lung involvement

Pulmonary vasculitis with diffuse alveolar haemorrhage is seen in a third of SSc-AAV patients, while up to 80% of patients develop ILD (often non-specific interstitial pneumonitis).^23,32^ The Australian cohort reported an independent association of ILD with SSc-AAV, after controlling for the ATA+,^22^ whereas, anti-PR3+ was not associated with ILD. There was a strong association of SSc-AAV and pulmonary emboli, particularly in anti-PR3+ patients. The increased prevalence of ILD in SSc-AAV compared to SSc alone is postulated to be secondary to subclinical vasculopathy.

Digital vasculopathy is present in 10% of cases, which ranges from ischaemic digital ulceration to a necrotizing vasculitis.^23^ Differentiating between the active vasculitis complicating SSc vasculopathy, or vasculopathy alone is challenging, but those with active inflammatory markers, or systemic presentations with renal or lung disease, should be managed as AAV.

Neurological involvement in the form of mononeuritis multiplex, and peripheral neuropathies is rarely reported in SSc, but complicates 15% of SSc-AAV cases.^32^

Observational studies have reported up to a 1.6-fold increased hazard of mortality in patients with SSc-AAV. The exact reason for this is unclear, but may be related to ILD, pulmonary emboli, or other organ involvement.

There is no evidence for a difference in frequency or presentation of cardiac or gastrointestinal involvement between SSc and SSc-AAV.^22^

## Management strategies

Differentiating the driver of clinical presentation is key to managing SSc-AAV patients. Given the risks of high dose steroids in early SSc, thorough investigations are required prior to their initiation.

For renal disease, renal biopsy is the definitive diagnostic tool. Prior to this, assessing the urine for haematuria, and renal casts suggests AAV. Proteinuria can be seen with both diseases; however, the protein leak tends to be less than 1 g/day in SRC. Renal biopsy is not required if the presentation is classical of AAV or SRC, thus should only be performed when presentations are clinically ambiguous.^10^

There is no specific treatment strategy for SSc-AAV, other than managing the underlying presentation. Treatment has included high-dose steroids, and cyclophosphamide, followed by maintenance therapy with azathioprine or mycophenolate mofetil. Rituximab has also been utilised, as has plasma exchange in advanced cases.^34^

Although this review has focussed on SSc-AAV, the reader must also investigate other causes of vasculitis in SSc, including cryoglobulinaemia, overlap with connective tissue diseases and secondary infections.

## Conclusion

Independently, both in AAV and SSc, there is increasing arguments that definitions of the diseases would be more accurate if the subgroups were classified by autoantibody subtype. This may allow more accurate risk assessment of organ involvement, as well as offering the potential for improved targeted treatments.

What remains to be assessed, is whether specific targeted treatment approaches might benefit overlap syndromes, especially as targeted biological agents, such as rituximab, which have been shown effective in both conditions.^34,35^ We know, however, that the majority of patients with overlap SSc-AAV tend to carry the ATA antibody and the MPO antibody, and the combination of these autoantibodies in SSc-AAV confers a significantly increased risk of ILD. Thorough assessment is crucial to ensure that treatment not only focusses on the most likely diagnosis driving pathology in SSc-AAV, but also improves outcome in this high mortality disease (Table 2).

**Table 2.** Glossary of terms. Terminology.

Antibody	Terminology	Remarks
SSc-AAV	Overlap systemic sclerosis and ANCA- associated vasculitis	
c-ANCA	Cytoplasmic antineutrophil cytoplasmic antibodies	Associated with anti-PR3
p-ANCA	Peripheral antineutrophil cytoplasmic antibodies	Associated with anti-MPO
GPA	Granulomatosis with polyangiitis	Majority PR3-ANCA +
MPA	Microscopic polyangiitis	Majority MPO-ANCA +
EGPA	Eosinophilic granulomatous polyangiitis	MPO-ANCA most frequent antibody identified
ATA	Anti-topoisomerase antibody	Most frequent antibody in dcSSc
ARA	Anti-RNA polymerase III antibody	Associated with dcSSc
Anti-MPO	Antibodies against myeloperoxidase	
Anti-PR3	Antibodies against proteinase-3	

ANCA: antineutrophil cytoplasmic antibodies; ATA: anti-topoisomerase; AAV: antineutrophil cytoplasmic antibody-associated vasculitis; PR3: proteinase; MPA: microscopic polyangiitis; GPA: granulomatosis with polyangiitis; SRC: scleroderma renal crisis; ILD: interstitial lung disease.
